# Toward traceable global systems for end-of-life photovoltaic waste

**DOI:** 10.1038/s41467-026-69171-z

**Published:** 2026-02-19

**Authors:** Beijia Huang, Yuqiong Long

**Affiliations:** Department of Environment and Architecture, University of Shanghai for Science and Technology, Shanghai, China

**Keywords:** Environmental impact, Sustainability

## Abstract

Rapid global expansion of photovoltaics is driving degraded module flows to emerging markets. This flow occurs amid limited regulatory oversight and recycling capacity, posing substantial environmental risks to importing regions. Mitigating these risks necessitates cross-border governance and traceable end-of-life systems.

The rapid growth of the photovoltaic (PV) industry has made it an important force in driving the global energy transition and carbon neutrality goals. The global installed PV capacity is expected to further increase to about 4500 Gigawatt (GW) by 2050^1^. China, the United States. India, Japan, and Germany are leading countries of PV module manufacturing^2^ (see Fig. 1b, c). As PV deployment expands worldwide, rapid technological progress in major manufacturing countries is also accelerating the replacement of older modules, generating substantial volumes of decommissioned or degraded PV modules. With limited resale value in mature PV markets, a large share of these degraded modules is redirected to secondary markets in emerging and developing regions^3^. Although the low-price importation of degraded PV modules has alleviated energy access challenges, their inherent performance limitations transfer operational and environmental burdens to importing nations. This increasing exposure to degraded PV modules creates emerging cross-border risks due to weak quality assurance, limited recycling capacity, and the potential accumulation of unmanaged PV waste.

**Fig. 1:**
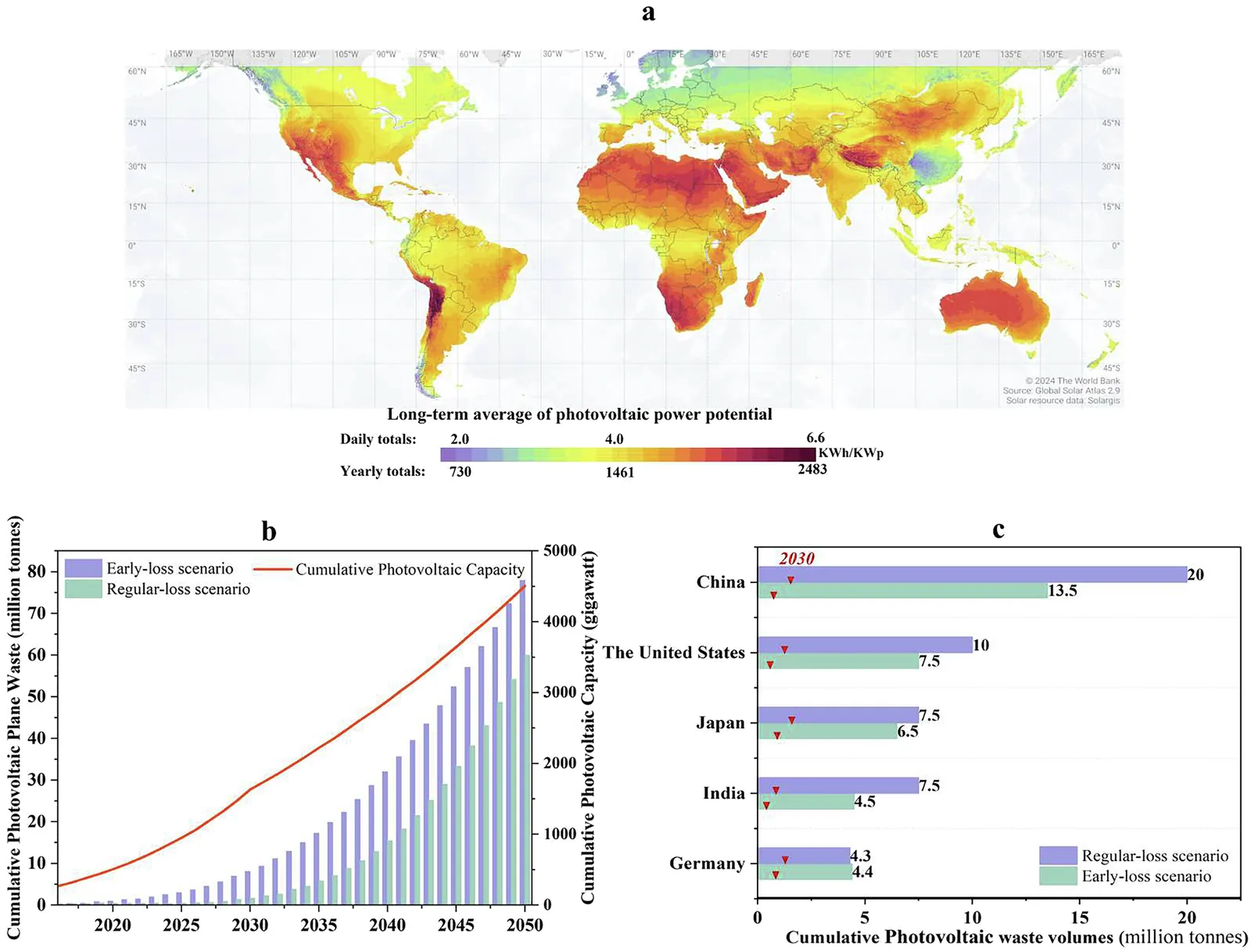
Global Photovoltaic installation potential and panel waste prediction. **a** Photovoltaic power potential^23^; **b** Global Photovoltaic capacity, and Panel waste^1^; and **c** Cumulative waste volumes of five countries (China, the United States, Japan, India, and Germany) of end-of-life Photovoltaic panels in 2050^1^.

## Growing flows of degraded PV modules under weak oversight

PV modules exhibiting cosmetic defects, unstable electrical performance, or operational degradation, thereby failing to meet established quality standards, are classified as degraded PV modules^4^. Degraded PV modules predominantly result from manufacturing defects, technological obsolescence, premature decommissioning, and operational failures^5^. Rapid advancements in PV technology and escalating efficiency standards in technologically advanced markets, such as China, the United States, Japan, and Germany, have rendered earlier-generation, lower-efficiency modules commercially obsolete. In these mature PV markets, earlier-generation low-power modules, typically below 400–500 watt-peak (Wp), have virtually no remaining market presence and now constitute a major source of degraded PV components^6^. Operational failures constitute a critical factor in the growing number of degraded PV modules. Additionally, approximately 70% of PV modules are decommissioned before reaching the end of their design lifespan (around 25-30 years)^7^. A considerable share of these prematurely retired modules is refurbished, typically through basic cleaning and electrical testing, and reintroduced into secondary markets. Many of these refurbished units are subsequently exported, often exhibiting 10–20% power degradation and inconsistent quality^8^.

Degraded PV modules are disseminated through three primary channels: direct trade, international aid programs, and bulk low-cost sales. These modules predominantly flow to Africa (including Nigeria, Kenya, and South Africa) and the Middle East (notably Saudi Arabia and the United Arab Emirates)^9^. However, most of these emerging markets lack mature quality assurance systems for imported PV equipment, including mandatory testing or performance certification. As a result, recycling regulations are fragmented, and mandatory certification protocols are inconsistently applied. Moreover, exporting nations have not yet established a comprehensive regulatory framework that covers the entire life-cycle of PV products. For instance, in Nigeria, the absence of formal e-waste legislation and a dedicated PV waste recycling framework has led to the uncontrolled accumulation of imported second-hand solar panels, with informal recyclers handling disposal using unsafe practices that release hazardous substances into the environment^10,11^. Similarly, South Africa, one of the world’s largest and fastest-growing PV markets with abundant solar resources (Fig. 1a), has yet to develop a comprehensive regulatory framework for the end-of-life management of solar PV products^12^. Collection and recycling are mainly conducted by private or pilot programs with limited traceability and no standardized reporting requirements. These examples highlight the fragmented and uneven regulatory landscape across PV markets, underscoring the urgent need for harmonized policy frameworks and infrastructure development to ensure environmentally sound PV waste management. Consequently, the cross-border movement of degraded PV modules operates under weak regulatory control and limited information transparency. Although exporters may label products as degraded, importers often lack sufficient information on their quality and associated environmental risks. In regions with high energy access costs, users tend to prioritize low initial investment over long-term environmental and maintenance costs.

## Environmental risk from improper disposal of PV modules

The export of degraded PV modules has precipitated multiple environmental challenges. Their elevated power degradation rates and reduced lifespans exacerbate environmental management burdens and complicate e-waste disposal. Many emerging and developing regions lack robust regulatory oversight, traceability of environmental liabilities, and policy support for second-hand modules, resulting in inadequate PV waste management. As export volumes increase, these issues become increasingly pronounced. The lack of recycling infrastructure and technical capacities (e.g., high-temperature pyrolysis and chemical etching) hinders the development of even basic systems for PV waste collection, sorting, and transportation of the increasing volume of discarded modules^11^. The PV waste is usually treated through land-filling and incineration, further exacerbating environmental degradation.

**Fig. 2:**
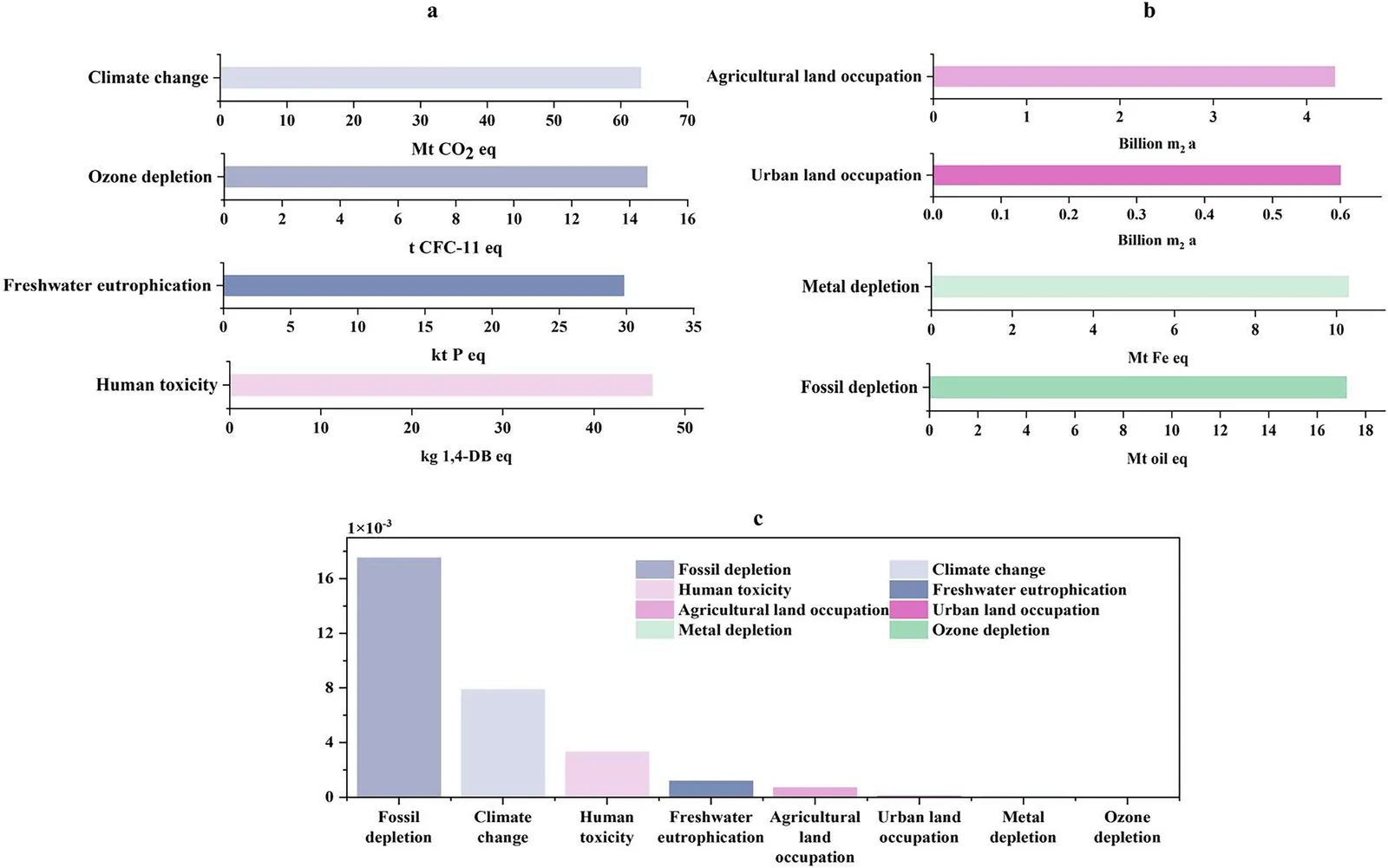
Environmental impacts of land-filling of photovoltaic waste in the Middle East and Africa in 2050. **a**, **b** refers to the key environmental impacts: climate change (Mt CO₂ eq), ozone depletion (tonnes of trichlorofluoromethane equivalents, t CFC-11 eq), freshwater eutrophication (kilograms of phosphorus equivalent, kg P eq), human toxicity (kilograms of 1,4-dichlorobenzene equivalents, 1,4-DB eq), agricultural land occupation (Billion m²·a), urban land occupation (Billion m²·a), metal depletion (Fe eq), and fossil resource depletion (kg oil eq); **c** shows the normalized key environmental impacts assessment results, calculated using the ReCiPe 2016 midpoint (H) method. These standardized indicators facilitate cross-category comparison of environmental burdens.

By 2050, Africa and the Middle East are expected to generate 1.6 million tonnes (Mt) and 1.7 Mt of PV waste modules, respectively, with over 90% originating from imports and accounting for failures at different stages, including infant, mid-life, and wear-out, before reaching the 30-year lifespan^1^. Under the assumption that end-of-life PV modules in these regions will be disposed of in landfills, a life-cycle impact assessment was conducted using the ReCiPe 2016 midpoint method. The results indicate that the primary environmental impacts are fossil energy consumption, climate change, and human toxicity (Fig. 2c). Land-filling PV waste is projected to consume metal resources equivalent to 10.3 Mt Fe and fossil resources equivalent to 17.2 Mt oil, increasing pressure on raw material extraction and exacerbating the ongoing energy and resource crisis^13^. In addition, land-filling PV waste is estimated to emit approximately 63 Mt of CO₂, thereby intensifying global climate change and undermining progress toward Sustainable Development Goal (SDG) 13 (Climate Action) (Fig. 2a).

Moreover, heavy metals and hazardous chemicals such as cadmium, selenium, and ethylene-vinyl acetate (EVA) can leach into soil and groundwater during land-filling, resulting in human toxicity equivalent to 4.64 Mt 1,4-dichlorobenzene (1,4-DB) and freshwater ecotoxicity equivalent to 1.1 Mt 1,4-DB^14^ (Fig. 2a). Such leaching of hazardous substances not only endangers groundwater safety and undermines access to clean drinking water and sanitation (SDG 6), but can also introduce selenium-related contaminants into aquatic and terrestrial food webs, where they may bioaccumulate and exert severe toxic effects, thereby increasing disease burden, shortening life expectancy, and contributing to premature mortality. Such impacts may trigger a severe public health crisis, contravening the objectives of ensuring healthy lives and promoting well-being for all (SDG 3)^15^. The land-filling of PV waste modules is projected to yield pollutants equivalent to 29.8 kilotonnes (Kt) of phosphorus-equivalent freshwater eutrophication agents, which may destabilize aquatic ecosystems, exacerbate algal blooms, and cause widespread mortality among aquatic organisms, potentially culminating in complete ecosystem collapse (Fig. 2a). Such pollutant-induced ecological disturbances pose a significant threat to marine and coastal ecosystems and biodiversity, thereby hindering the sustainable management of marine resources (SDG 14).

Meanwhile, the non-biodegradable nature of these materials results in the long-term occupation of urban and agricultural lands, estimated at 0.6 billion square metres per year (m^2^a) and 4.3 billion m^2^a, respectively, thereby reducing land-use efficiency and constraining space for ecosystems and food production, with ecological restoration potentially requiring decades to centuries (Fig. 2b). Additionally, the release of 14.6 t trichlorofluoromethane equivalents (CFC-11) of ozone-depleting substances undermines the ozone layer, increasing harmful ultraviolet radiation and compromising global ecological safety (Fig. 2a). Given that PV waste also contains substantial quantities of recyclable resources and critical metals such as molybdenum, magnesium, gallium, and indium, improper disposal poses a long-term threat to environmental management and sustainable development, underscoring the urgent need for accelerated recycling and resource recovery.

## Actions for sustainable management of degraded PV modules

### Establishing clear governance frameworks across exporters, importers, and international institutions

Effective management of degraded PV modules requires a coordinated governance framework that differentiates the responsibilities of exporting countries, importing countries, and international institutions. *Exporting countries* should provide transparent documentation and ensure compliance with applicable international standards, such as International Electrotechnical Commission (IEC) 61215 series (Terrestrial photovoltaic modules – Design qualification and type approval) and IEC 61730 series (Photovoltaic module safety qualification), which certify module performance, durability, and environmental safety before shipment. These documents should disclose essential upstream information, including manufacturing specifications, material composition, warranty and testing records, and any refurbishment or repair history, thereby enabling importing countries to verify product quality and assess potential environmental risks. Such information helps prevent the transfer of low-quality or environmentally hazardous products into markets with limited regulatory capacity. *Importing countries* should establish pre-import testing, independent certification systems, and regulatory mechanisms that verify module integrity, energy efficiency, and expected lifespan. Governments can further introduce fiscal incentives and deposit—refund mechanisms to support licensed recyclers and encourage the development of domestic recycling industries. At the international level, establishing coordinated governance and clear responsibility mechanisms is essential to prevent unmanaged end-of-life flows of degraded PV modules from becoming major sources of environmental leakage. Drawing on life-cycle assessment findings, frameworks such as Germany’s business-to-consumer (B2C) and business-to-business (B2B) frameworks can clarify stakeholder responsibilities and ensure equitable cost-sharing for recycling. The implementation of extended producer responsibility (EPR) can further require manufacturers to retain post-export collection and disposal obligations^16^, thereby improving material efficiency and promoting responsible waste management in alignment with SDG 12. In many emerging and developing regions, recycling industries remain nascent, constrained by limited financial resources, inadequate facilities, and technological gaps. Hence, multilateral cooperation is essential to provide technical assistance, concessional financing, and targeted recycling incentives. Lessons from the European Union’s Waste Electrical and Electronic Equipment (WEEE) Directive -based EPR system, including mandatory producer registration, centralized collection schemes, and standardized reporting requirements, offer practical policy models for importing regions such as the Middle East and Africa to establish traceable, regulated end-of-life pathways for their own PV waste management frameworks. Extending existing e-waste recycling operations to incorporate PV modules would enhance processing efficiency, lower costs, and promote a globally coordinated PV recycling network^17^. A standardized global trading system for recycled PV modules and materials, integrated within a unified life-cycle management framework, can bridge policy gaps, improve resource efficiency, and advance recycling industry professionalization.

### Building transparent and interoperable data systems for global traceability

A transparent, reliable global database for PV modules is essential for tracking degraded PV modules throughout their life cycle and ensuring accountability in transboundary movements. The immediate priority is to establish standardized, internationally accessible databases that record module origin, type, composition, and estimated service life. *Exporting countries* should be mandated to disclose these data through harmonized reporting platforms verified by third-party agencies, while *importing countries* should use the information to improve customs screening, verify product authenticity, and align national recycling capacity with actual inflows. In the long term, *international organizations* such as the International Renewable Energy Agency (IRENA) and the International Organization for Standardization (ISO) are encouraged to coordinate the development of unified classification systems, data formats, and verification protocols. The main challenge lies not in the availability of digital technologies but in the absence of consistent reporting standards, transparent data sharing, and reliable verification mechanisms. Therefore, near-term efforts should focus on building open-access databases, unified reporting formats, and cross-border information exchange protocols to eliminate data asymmetry between exporting and importing countries. Advanced digital tools, such as Internet of Things tagging and blockchain, can later complement these systems once a solid data infrastructure is established^18^. Pilot initiatives such as the SolarCoin^19,20^ renewable energy certification program and the UNDP-supported Blockchain for Sustainable Supply Chains project^21^ have demonstrated the potential of blockchain-based systems^22^ to enhance transparency and traceability in renewable energy and e-waste management. However, large-scale deployment remains unrealistic for most developing countries due to limited digital capacity, high system costs, and fragmented governance frameworks. Prioritizing fundamental data standardization and transparent reporting will thus provide the institutional foundation for future digitalization of PV waste management. While these governance measures primarily aim to reduce environmental risks, they also involve economic and social trade-offs. Stricter export controls and higher recycling standards may increase compliance costs, potentially influencing energy affordability and access in importing countries, particularly in regions with limited financial capacity. Balancing environmental integrity with economic feasibility and social equity is therefore essential for the effective implementation of sustainable PV module management.

### Promoting modular and recyclable PV module design

Promoting Modular and Recyclable PV Designs through Manufacturer, Regulator, and Standards Body Collaboration. At the industry level, PV manufacturers should prioritize reduced resource consumption and lower material intensity to maximize product durability and optimize existing assets. Standardizing module materials with an emphasis on environmentally benign alternatives and easily disassembled structures will facilitate efficient end-of-life recycling. Reducing toxic material use, adopting recyclable encapsulants and adhesives, and developing novel lead-free solders can substantially lower waste management costs. At the policy level, exporting and importing governments should adopt clear eco-design standards and provide fiscal or regulatory incentives to encourage the adoption of recyclable and low-toxicity materials. International organizations such as the ISO and the IRENA could facilitate the development of harmonized technical standards and promote cross-border technology transfer. Beyond design and regulatory standards, effective end-of-life management will depend on scaling up advanced dismantling and recovery technologies through coordinated action among public authorities, industry, and research institutions. Collectively, these measures can close material loops, increase the reintegration of secondary raw materials into production, and accelerate the transition toward a circular and low-carbon PV industry.

## Conclusions

Despite notable international endeavors to address PV waste management, existing governance frameworks remain insufficient to mitigate the rising transboundary risks associated with degraded PV modules. Addressing this challenge requires coordinated action among exporting and importing countries, as well as international organizations, to promote circular design, effective recycling, and environmentally sound waste management. We advocate for establishing a recycling framework for degraded PV modules, enhancing resource recovery efficiency, and promoting innovative, environmentally sound recycling technologies as core elements of a circular PV economy. Leading PV producing and consuming nations should adopt equitable, transparent, and sustainable waste management practices. These initiatives can strengthen the sustainability of the global PV industry while supporting Affordable and Clean Energy (SDG 7) and Sustainable Cities and Communities (SDG 11), particularly in regions with limited power supply. Future research should focus on quantifying the environmental and economic benefits associated with the reuse of degraded PV modules, evaluating their transboundary environmental impacts, and formulating region-specific strategies to optimize resource-efficient material recovery.
